# Wearable Conductive Fiber Sensors for Multi-Axis Human Joint Angle Measurements

**DOI:** 10.1186/1743-0003-2-7

**Published:** 2005-03-02

**Authors:** Peter T Gibbs, H Harry Asada

**Affiliations:** 1 Department of Mechanical Engineering, Massachusetts Institute of Technology, 77 Massachusetts Ave. 3-351, Cambridge, Massachusetts, USA

## Abstract

**Background:**

The practice of continuous, long-term monitoring of human joint motion is one that finds many applications, especially in the medical and rehabilitation fields. There is a lack of acceptable devices available to perform such measurements in the field in a reliable and non-intrusive way over a long period of time. The purpose of this study was therefore to develop such a wearable joint monitoring sensor capable of continuous, day-to-day monitoring.

**Methods:**

A novel technique of incorporating conductive fibers into flexible, skin-tight fabrics surrounding a joint is developed. Resistance changes across these conductive fibers are measured, and directly related to specific single or multi-axis joint angles through the use of a non-linear predictor after an initial, one-time calibration. Because these sensors are intended for multiple uses, an automated registration algorithm has been devised using a sensitivity template matched to an array of sensors spanning the joints of interest. In this way, a sensor array can be taken off and put back on an individual for multiple uses, with the sensors automatically calibrating themselves each time.

**Results:**

The wearable sensors designed are comfortable, and acceptable for long-term wear in everyday settings. Results have shown the feasibility of this type of sensor, with accurate measurements of joint motion for both a single-axis knee joint and a double axis hip joint when compared to a standard goniometer used to measure joint angles. Self-registration of the sensors was found to be possible with only a few simple motions by the patient.

**Conclusion:**

After preliminary experiments involving a pants sensing garment for lower body monitoring, it has been seen that this methodology is effective for monitoring joint motion of the hip and knee. This design therefore produces a robust, comfortable, truly wearable joint monitoring device.

## Background

Long-term measurement of human movement in the field is an important need today [1]. For many types of rehabilitation treatment, it is desirable to monitor a patient's activities of daily life continuously in the home environment, outside the artificial environment of a laboratory or doctor's office [2]. This type of monitoring is quite beneficial to the therapist, allowing a better assessment of human motor control, and tremor or functional use of a body segment, over long periods of time [1]. Evaluating a patient's daily life activities allows a more reliable assessment of a patient's disabilities, and aids in developing rehabilitation treatments and programs, as well as assessing a treatment's effectiveness [2,3]. In addition, the recognition of deviations in joint movement patterns is essential for rehabilitation specialists to select and implement an appropriate rehabilitation protocol for an individual [4,5].

Many specific medical applications benefit from the information provided by continuous human movement monitoring. To better develop and optimize total joint replacements, for instance, a detailed record of a patient's daily activities after such a replacement is required [6]. The measurement of tremor and motor activity in neurological patients has long been studied [7]. In pulmonary patients, it is often desirable to precisely quantify the amount of walking and exercise performed during daily living, since this is a fundamental goal in improving physical functioning and life quality [3]. Furthermore, physiological responses, such as changes in heart rate or blood pressure, often result from changes in body position or activity, making the assessment of posture and motion an essential issue in any type of continuous, ambulatory monitoring [8].

Presently, there is no satisfactory solution for long-term, human movement monitoring in the field. The use of video and optical motion analysis systems offer the most precise evaluation of human motion, but obviously restrict measurements to a finite volume [9]. Body mounted sensors such as accelerometers and pedometers are used for monitoring daily physical activity, but those devices are unable to detect the body posture and are often limited in reliability and applicability [3,7]. Even methods of self-report designed to gather information on general daily activity, such as diaries or questionnaires, are time consuming and often unreliable, especially for the elderly relying on their memory [3].

Electrogoniometers are frequently used to measure dynamic, multi-axis joint angle changes in individuals, providing continuous joint movement information. These devices, however, are not desirable for long-term monitoring of daily living, since they are exoskeletal devices that cross the joint, potentially interfering with movement. Furthermore, any shift from their original placement leads to errors in angle estimations [2]. Such commercially available goniometers can produce erratic readings once the device is detached from the patient body and put back on the same joint in a slightly different orientation. It is therefore difficult to use these goniometers at home for long periods of time.

Other types of goniometric devices have been developed for measuring particular parts of the body. Electronic gloves [10-13], for example, can measure the hand posture accurately, but are often cumbersome to wear for long periods of time. Various types of textile fabrics with integrated sensing devices have also been devised [14,15]. In each of these cases, the sensing devices are traditional strain gauges, carefully attached to an article of clothing. One patented device uses conductive fabrics acting as strain gauges on a garment to emit "effects" such as light or sound based upon a wearer's movements [16]. While this is a novel wearable device, it is not designed, nor is suitable, for long-term accurate joint angle measurement.

For all types of body-mounted sensors, the issues of comfort and wearability are of major importance, if a patient has to wear the monitoring device for extended periods of time. Furthermore, such home-use wearable sensors need to be put on and off every day without close supervision of a medical professional. Proper registration of the sensor is therefore a crucial requirement for deploying wearable sensors to the home environment.

The goal of this paper is therefore to develop a new method for continuous monitoring of human movement by measuring single or multi-axis joint angles with a wearable sensing garment that is non-intrusive and non-cumbersome and that can be properly registered for reliable monitoring. A new method is presented here for joint monitoring using conductive fibers incorporated into comfortable, flexible fabrics. All that is needed is a one-time calibration with a standard goniometer, and a conductive fiber sensor garment is then able to continuously detect joint movement and measure specific single or multi-axis joint angles. With an array of sensors incorporated into a sensing garment, registration of the sensor occurs automatically each time the garment is worn through only a few simple motions by the wearer. This type of wearable sensor would allow extended home monitoring of a patient, and is no harder to put on than a typical article of clothing.

In the following, the principle and design details of this wearable device will be presented, along with effective algorithms for allowing a patient to perform long-term, unsupervised monitoring in the home environment. Experimental feasibility tests will also be presented on a prototype wearable sensor for both single-axis and multi-axis joints.

## Methods

### Working Principle

The basic principle behind the wearable sensors presented in this paper is as follows: when a particular joint on the human body moves, skin around the joint stretches, along with any clothing surrounding the joint as well. A former study by the textile industry has shown that body movements about joints require specific amounts of skin extension. Lengthwise across the knee for example, the skin stretches anywhere from 35–45% during normal joint movement [17].

When a particular joint moves, fabric around the joint will either expand or contract accordingly, assuming the fabric is form fitting to the skin, and has the necessary elastic properties. To assure comfort and freedom of body movement, stretchability of 25 to 30 percent is recommended for fabrics fitting closely to the body [17]. By incorporating conductive fibers into such a fabric surrounding a joint, the conductive materials will necessarily change length with joint movement. The electrical resistance of the conductive material will change as well, and can be directly measured and correlated to changes in the orientation of the joint.

**Figure 1:**
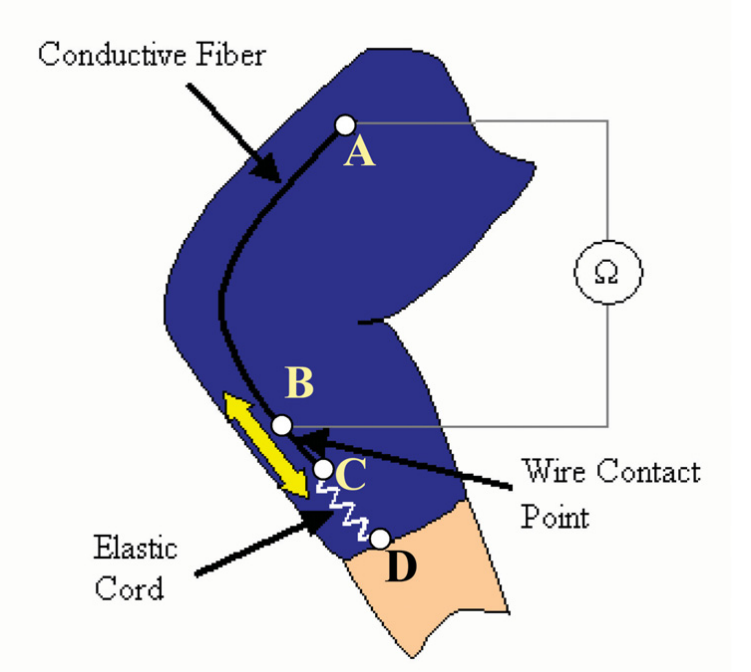
**Sensor Design Schematic. **This particular sensor arrangement shows one sensor thread running lengthwise across a single-axis knee joint.

Figure 1 shows how a single conductive fiber is implemented as a sensor. One end of the conductive fiber is permanently attached to the nonconductive, form-fitting fabric substrate at point *A *in the figure. Along the conductive fiber, there is a wire contact point at B that is permanently stitched into the fabric. The other end of the conductive fiber, point *C*, is kept in tension by a coupled elastic cord, which is permanently attached to the remote side of the joint, point *D*. Therefore, any stretching in this coupled material will take place in the highly elastic cord, *CD*, and not in the conductive fiber *AC*. As the joint moves, the elastic cord will change length, causing the coupled conductive fiber to freely slide past the wire contact point at *B *that is stationary. The conductive fiber always keeps an electrical contact with this wire, but the length of conductive thread between points *A *and *B *will change as the joint rotates. The resistance, which is linearly related to length, is then measured continuously across these two points *A *and *B*.

### Predictor Design

**Figure 2:**
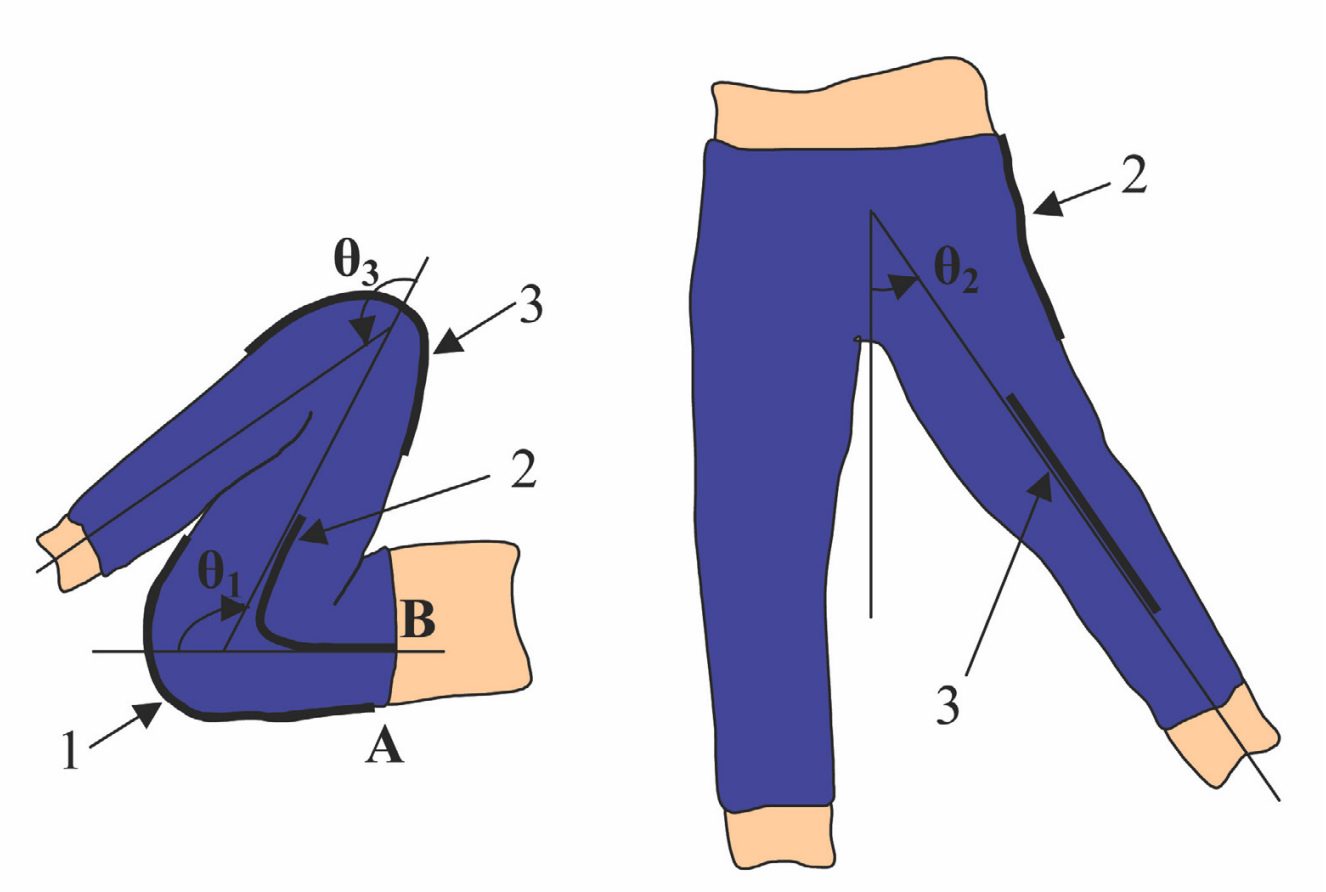
**Lower Body Sensors. **Schematic of three sensors positioned to measure three lower body joint angles.

Consider Figure 2. Shown here are a sensor spanning across a single axis knee joint, and a pair of sensors about a double axis hip joint. The angles of interest are labeled *θ*_1_, *θ*_2_, and *θ*_3_. Our goal is to estimate these joint angles based upon the output of sensors 1, 2, and 3.

**Figure 3:**
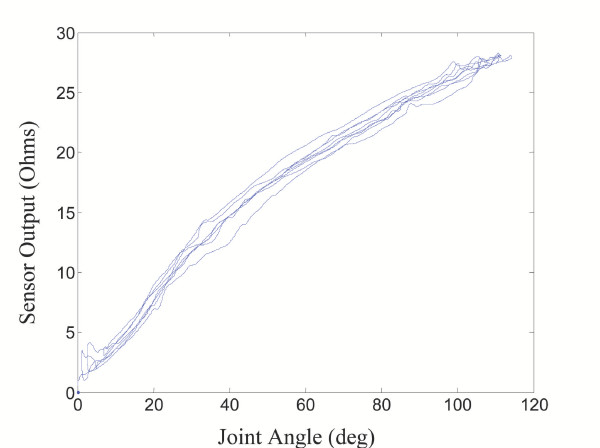
**Sensor Output Curve. **Preliminary data showing sensor output vs. knee flexion angle.

Preliminary experiments have shown a clear relationship between joint angle and sensor output for individual sensors about various joints of the body. Figure 3, for instance, shows a typical set of output data from a single sensor thread across a single-axis knee joint with the output "zeroed" for a joint angle of 0°.

It is desired to design a filter that receives sensor signals as inputs, and predicts the joint angle(s) of interest. In the proposed method, each joint angle being monitored has a corresponding single sensor that is situated about that particular joint for maximum sensitivity, as in Figure 2.

Consider *N *axis sensors for measuring *N *joints, each consisting of a single thread sensor, as shown in Figure 2. The simplest predictor model that can be used is a linear regression:



where  is the *N *× 1 vector of *N *joint angle predictions,  is its bias term, **y **= (*y*_1 _… *y*_*N*_)^*T *^is the *N *× 1 vector of corresponding sensor readings, and **G **and  are, respectively, the *N *× *N *matrix and the N × 1 vector experimentally determined to relate the inputs and the outputs.

Since there is a slight amount of curvature in the preliminary data of Figure 3, a nonlinear predictor may be more effective. We will use a second order polynomial model



where



and **G**' is an *N *× *N*(*N*-1)/2 experimentally determined matrix. The three terms on the right hand side of the above equation can be incorporated into a homogeneous expression using augmented matrix and vector:



where **W **and **Y **are

**W **= **(θ**_**0 **_**G G') **    (5)



To determine the parameter matrix **W**, a least squares regression is performed using *m *sets of experimental data from a collection of sensors on an individual patient. Let **P **be a *N *× *m *matrix consisting of *m *sets of experimentally measured joint angles,



and **B **be a {1 + *N*(*N *+ 1)/2} × *m *matrix containing the corresponding sensor outputs and their quadratic terms:

**B **= **(Y**^(1) ^… **Y**^(*m*)^)     (8)

The optimal regression coefficient matrix W* that minimizes the squared prediction errors is given by

W* = **PB**^**T **^**(BB**^**T**^**)**^-1 ^    (9)

if the data are rich enough to make the matrix product **BB**^**T **^non-singular.

The above expressions are the most general forms for *N *axis sensors. In practice, however, they can be reduced to a compact expression with lower orders. First the offset  can be eliminated from the coefficient matrix **W**, if the sensor outputs are zeroed at a particular posture, e.g. the one where the extremities are fully extended. Second, although the matrix **G **contains off-diagonal elements representing cross couplings among multiple joints, some joints have no cross coupling with other joints. For example, the measurement of the knee joint can be performed separately from that of the hip joints. If the *j*-th joint is decoupled from all others, it can be treated separately as:



where the offset is eliminated. Third, although multiple joints are coupled to each other having non-zero, first-order off-diagonal coefficients in matrix **G**, their second-order cross coupling terms, e.g. *y*_*j *_*y*_*k*_, can be negligibly small with proper design of individual sensors. In such a case, two coupled joints, say *j *and *k*, can be written as:



where the offset terms have been eliminated. Thus the number of parameters to identify through calibration experiments is reduced. In consequence, the dimension of the optimal coefficient matrix must be reduced accordingly. The same calibration procedure is performed for both single axis and multiple axis cases, and need be performed only once for a specific set of sensors on an individual.

Although one sensor is sufficient to capture single-axis joint motion, any misalignment of such a sensor from use to use will lead to erroneous measurements. From a practical standpoint, it is obvious that a method is needed to adjust for any shifting of a sensor about the joint that will take place from one use to the next. It is both undesirable and impractical to recalibrate the whole sensor every time the patient takes off the sensing garment and places it back again. To take care of such registration problems, an array of multiple sensor threads is used. By incorporating multiple threads in a known pattern, a template-matching algorithm can be performed to determine a sensor's offset from calibration. In this way, measurement errors due to sensor misalignment are significantly reduced. The details of this method are described in the next section.

### Sensor Registration for Single Axis Joints

The goal in designing these wearable sensors is to create a device that is ultimately self-registering for subsequent uses after the initial one-time calibration experiments. This means that no additional equipment is needed to register the sensors for each use. Also, it is important that any procedures that are needed for self-registration are simple, and able to be preformed by the patient without supervision. To achieve these goals, a multi-thread sensor array design is presented.

**Figure 4:**
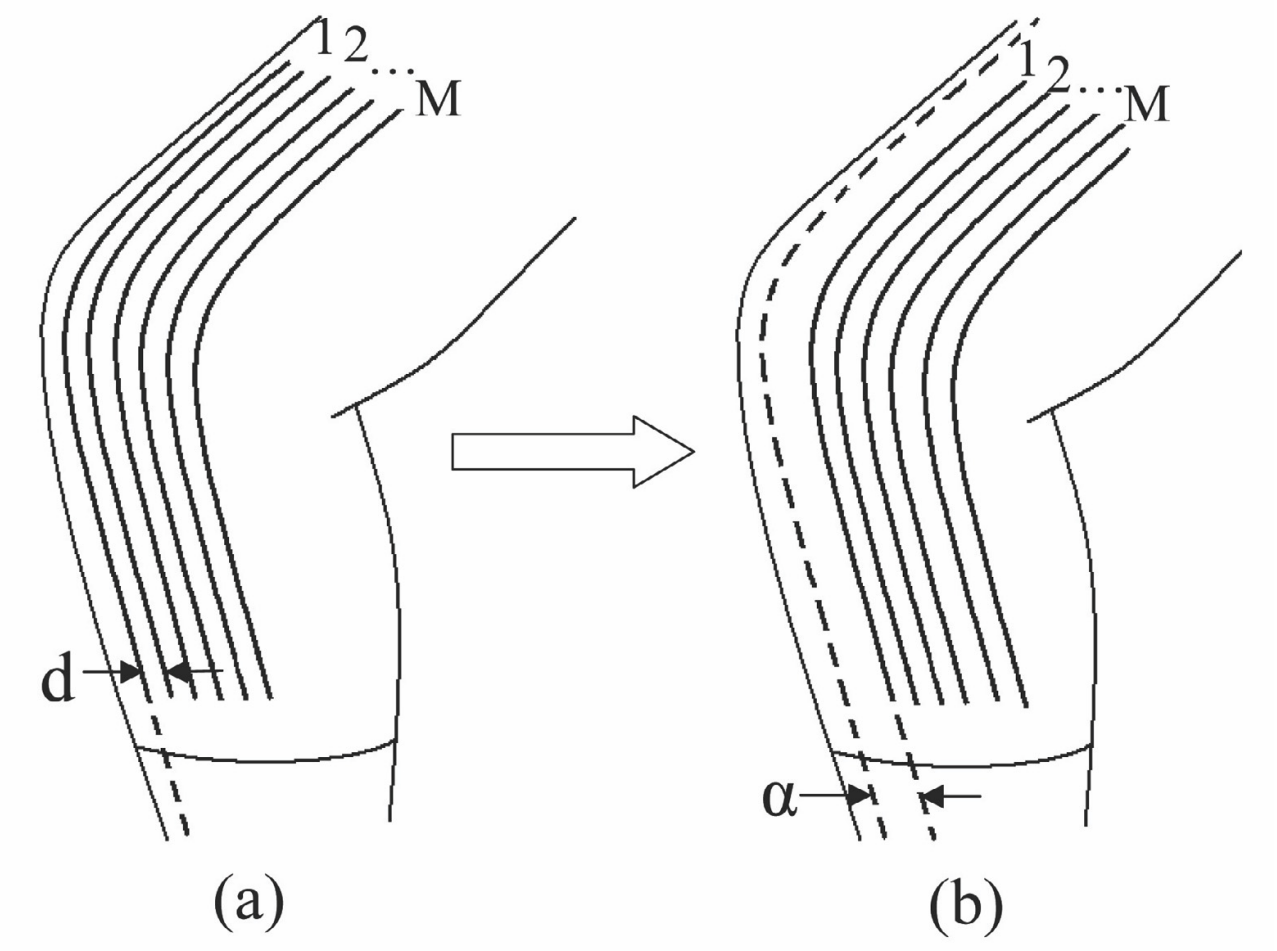
**Sensor Arrays. **(a) Array of equidistantly spaced sensors over knee joint. (b) Array shifted by an unknown distance, *α*.



First, consider an array of *M *sensors covering a single-axis joint as shown in Figure 4(a). Each sensor thread is separated from the adjacent sensor thread by a known, constant distance, *d*. This multi-thread sensor array is used to estimate a single-axis joint angle, *θ*_*j*_. To develop a registration procedure let us first calibrate each sensor thread individually. Let  be the estimate of the *j*-th joint based on the *i*-th thread sensor given by

where



and  is the 1 × 2 regression vector that is optimized for the *i*-th single-thread sensor of the *j*-th joint placed at a home position.

**Figure 5:**
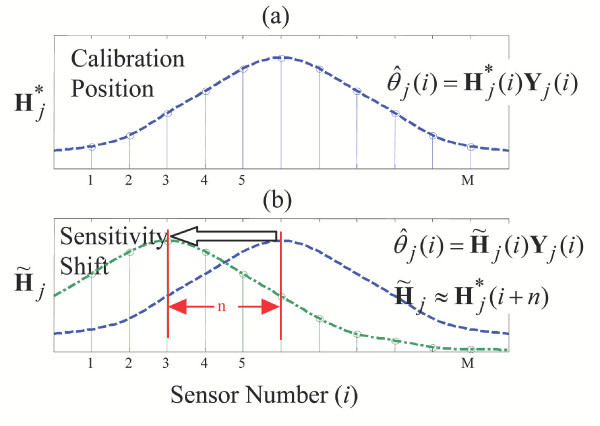
**Sensitivity Shifts. **(a) Array of equidistantly spaced sensors over knee joint, with each sensor having unique sensitivity in this calibration position. (b) Shifting of array by an unknown distance, *α*, will lead to a shift in sensitivities.

Now consider the situation where the sensor array has been removed, and placed back on the joint for more measurements. The sensor array is now offset an unknown distance, *α*, from the original position where calibration was performed. See Figure 4(b). Since the individual single-thread sensors in the array are equally spaced, each sensor thread is shifted from its home calibration position by the same distance *α*. Assuming that the individual sensor threads are identical other than being separated by a distance *d*, we can conclude that the pattern of the sensitivity array is a shifted version of the calibrated one, as shown in the simplified plots of Figure 5. This reduces the self-registration problem to a type of pattern matching problem.

 will no longer be the appropriate regression matrix to estimate *θ*_*j *_from **Y**_*j*_(*i*). A new, unknown vector  will instead relate the sensor output to *θ*_*j*_:



Although  is unknown, each individual sensor in the array should ideally give the same estimate for the actual joint angle at any time, so that



If the shifting of the sensor array were to happen in a discrete fashion,

*α *= *nd *    (16)

where *n *is an integer value, it is seen that



Since *n *is an unknown, it is desired to find an *n *that satisfies (15) and (17), rewritten as





where *n *is assumed to be |*n*| <*M *- 1. Namely, the sensor array, although shifted, can still cover the joint, having an overlap with the original sensor at the home position.

In the ideal, theoretical case, there will exist an integer *n *that can be found to exactly solve (18). Unfortunately, for practical usage, *n *will not be a discrete integer. Furthermore, *n *cannot be explicitly found since process and measurement noise will cause the sensor outputs to deviate from their "ideal" values. With the knowledge of  for *i *= 1 ~ *M*, however, it is possible to find the optimal integer *n *that *best *solves (18).

Let us first define the average joint angle estimate for M threads of sensor outputs for a given integer *n *as follows (with **Y **and H* reducing to scalars for the linear case):





The best estimate for *n *is found by minimizing the average squared error between each sensor's estimate and the average estimate with respect to *n *(i.e. reducing the variance in the estimated angle as a function of *n*):







Equations (20a) and (20b) are solved for *n *= -*M*+2, -*M*+3, ..., *M*-3, *M*-2. The value for  found from (20c) is then used in (17) to approximate each sensor's predictor regression matrix for this new offset position of the array. In the ideal discrete case, where *α *= *n*_*o*_*d*, *n*_*o *_is the discrete offset of the sensor array,  = *n*_*o*_, and *R*_*j*_() = 0.

For the non-ideal case, where *a *is not a discrete multiple of *d*, the minimum variance is not zero, *R*_*j *_() ≠ 0, but it will decrease as *M *increases, and *d *decreases.

Creating a denser sensor array in this way leads to more accurate estimates of sensor sensitivities, which in turn leads to more accurate estimates of *θ*_*j*_. Furthermore, since  can always be approximated using this algorithm, a one-time calibration is all that is needed for these wearable sensors to be used by a patient.

The registration algorithm takes place in real time as the sensor is in use. All that is needed for a patient to begin using these sensors is to first "zero" the sensor output with the joint fully extended in the 0° position, and then freely move the joint to obtain non-zero data. This non-zero data will then allow the self-registration to take place. While registration is not needed at all times, it should be performed during initial operation until an appropriate  is converged upon. Again, the denser the array of sensors used, the better the estimate obtained. Following this, the algorithm need not be performed as often, as long as the sensor array remains stationary for an individual use. To begin monitoring, it is assumed that  = 0.

### Sensor Registration for Double Axis Joints

In the double axis case, two sensor arrays are placed around a predominantly two-axis joint such as the hip. As in the single-axis case, each array contains M sensor threads equally spaced by a distance *d*. The *j*-th joint array is placed so that it is most sensitive to changes in *θ*_*j*_, while the *k*-th joint array is situated so that it is most sensitive to changes in *θ*_*k*_.

**Figure 6:**
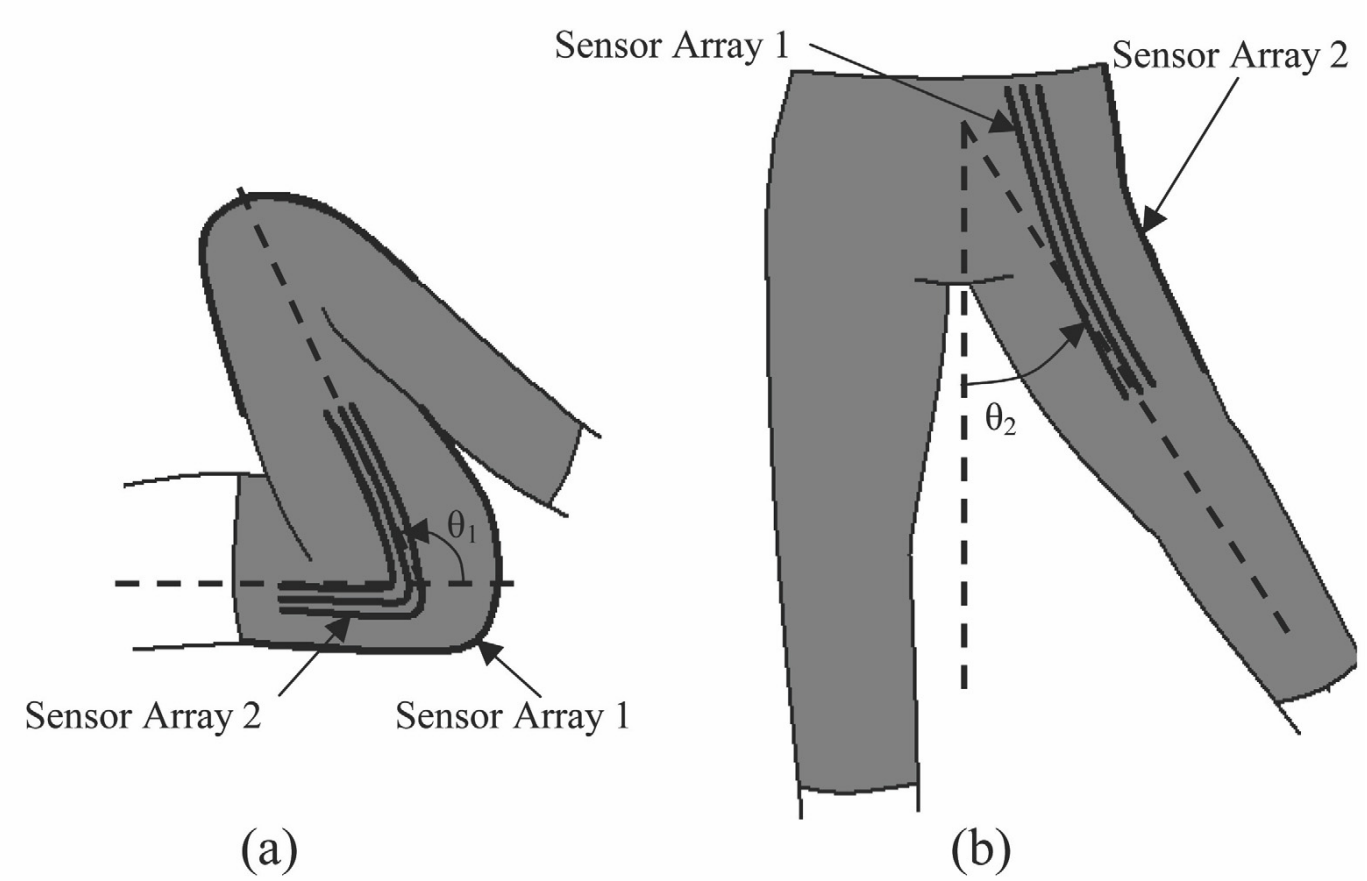
**Registration Procedure for Hip Sensor Array. **For registration of individual sensor arrays, the patient moves only one axis at a time (a) flexion/extension, and (b) abduction/adduction.

For registration, let the patient move only one axis at a time. As illustrated in Figure 6-(a), the patient is instructed to move axis *θ*_1 _alone. This hip flexion/extension causes significant changes to sensor array 1, *y*_1_(*i*), *i *= 1 ~ *M*. Next the patient is instructed to make hip abduction/adduction (*θ*_2_) alone, which causes significant changes to sensor array 2, as shown in Figure 6-(b). Until registration has been completed, the estimate of the joint angles is not accurate. However, it is possible to distinguish which joint, *θ*_1 _or *θ*_2_, has been moved, since sensor array 1 is most sensitive to *θ*_1_, and sensor array 2 for *θ*_2_. Once the individual axis movements are detected, the same registration procedure as that of a single axis can be applied to determine the misalignment of each sensor array. Once the misalignment is determined, the corrected, optimal predictor can now be used for verifying whether the registration has been performed correctly based on individual axis movements.

This registration method reduces the multi-axis problem to individual single axis procedures. However, the single axis procedures do not have to be repeated for all axes, if they are tightly related. For the two hip axes in Figure 6, a shifting of one sensor array around the body will be accompanied by a nearly identical shift in the second array. Therefore, registering one array will also register the other. In this case, it is required that a patient performs only one simple movement when first putting on the sensors – extending the joint about a single axis over a sufficient range.

## Results

All experiments have been conducted under a protocol approved by the Massachusetts Institute of Technology Committee on the Use of Humans as Experimental Subjects (Approval No. 0411000960).

### Wearable Prototype Garment

**Figure 7:**
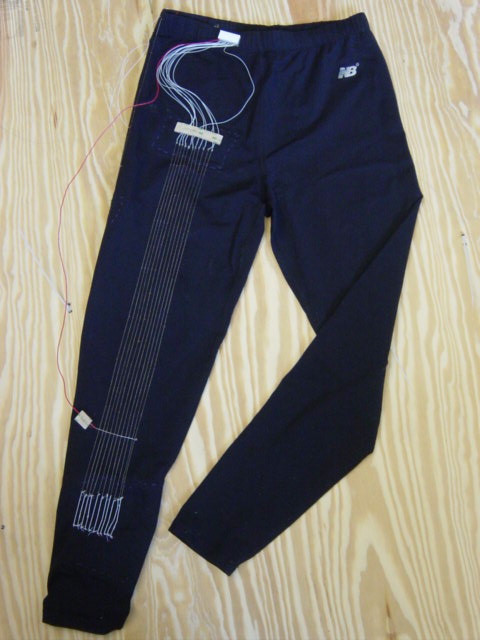
**Prototype Sensing Garment. **Spandex pants with conductive fiber sensors for lower body monitoring.

Figure 7 shows a prototype pair of spandex pants with conductive fibers incorporated into the fabric to measure lower body movement. Spandex was chosen due to its favorable qualities: very stretchable, elastic, fits closely to the skin, and is able to withstand normal body movements and return to its original shape with no permanent distortions [17]. Furthermore, it is a comfortable material, able to be worn on a daily basis since it does not restrict movement in any way. Thus it is quite suitable for this sensor design.

In these particular pants, an array of eleven sensors spans across the knee joint, each separated by a distance of 5 mm, and each with an unstretched length of 55 cm. The sensors threads were silver plated nylon 66 yarn, which had an impedance of approximately 3.6 Ω/cm. Single sensors span both the posterior and side of the hip as well to capture two axes of hip motion. These single sensors are not seen in the view of Figure 7, but the locations are the same as those shown for sensors 1 and 2 in the schematic of Figure 2. This is the sensing garment used for all experimental tests.

### Preliminary Experiments

**Figure 8:**
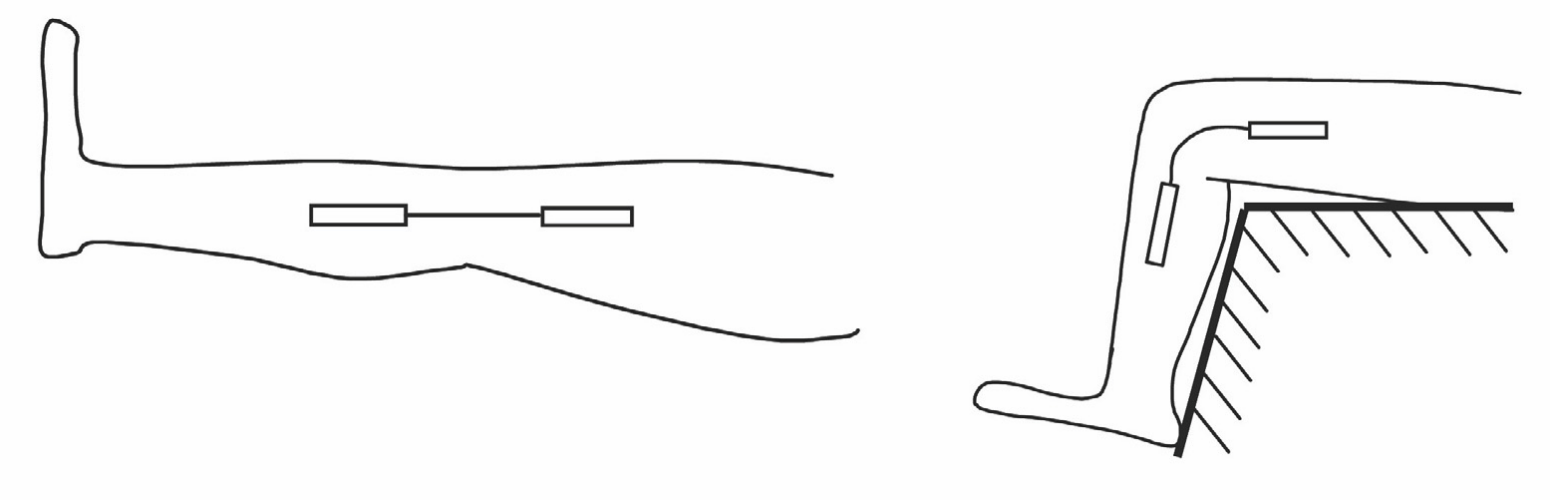
**Preliminary experiments set-up. **Measurements were taken from a standard electrogoniometer at Position 1 (0°) and Position 2 (50°)

To get an idea of the capabilities of existing technology available for joint monitoring, tests were initially performed using a standard electrogoniometer. Figure 8 shows the set-up of the preliminary experiments. The goniometer used was a BIOPAC TSD130B Twin Axis Goniometer that consisted of two telescoping end-blocks that were taped to the side of the leg on either side of the knee joint. A strain gauge between these blocks was the device that measured the joint angle. The goniometer was used to measure knee flexion angle for two discrete positions. An untrained professional attached the goniometer to the leg, but followed the recommended attachment procedures as described by the vendor in the instruction manual. This was to simulate the knowledge of a typical patient who would be using such a device on his or her own, outside a carefully controlled setting.

The goniometer was taken off and placed back on the knee joint eight separate times. Each time the goniometer was put on, the leg was extended (Position 1) and the goniometer output was set to 0°. The leg was then slowly bent to Position 2 (50°) and the goniometer output was recorded. The average rms error between the goniometer output, and the known joint angle (50°) for these tests was 3.5° with a standard deviation of 2.6°. Even with the goniometer placed on the same joint by the same person, these results illustrate the fact that slight changes in how the goniometer is attached can lead to varying measurements. It will be important to keep errors such as these in mind when the results from the conductive fiber sensor are analyzed.

Having just discussed the possible errors introduced by a standard electrogoniometer, it is important to also highlight the possible errors introduced by a conductive fiber thread sensor. Consider again Figure 3, which shows sensor output vs. knee flexion angle for one thread sensor on the pants garment when the knee was randomly swung over a large range of motion.

As can be seen, there is a significant amount of variation possible in sensor output for a given joint angle. In particular, for threads over the knee joint, the average rms error between curves such as those shown in Figure 3, and the calibrated predictor curves from (10) was approximately 3°-5° over the many tests performed. Therefore, it is noted upfront that errors will be introduced based solely on the type of measuring device being used due to hysteresis, material uncertainties, and other processes that cannot be accurately modeled. This should be kept in mind when using such a wearable device.

### Single Axis Results

The pants sensing garment was first used to estimate single-axis knee angle measurements. For the following single-axis experiments, a rotary potentiometer firmly attached to the leg was used as a goniometer, and this was the standard for which to compare joint angles. In each experiment, the potentiometer was "zeroed" with the leg in the full extension position.

**Figure 9:**
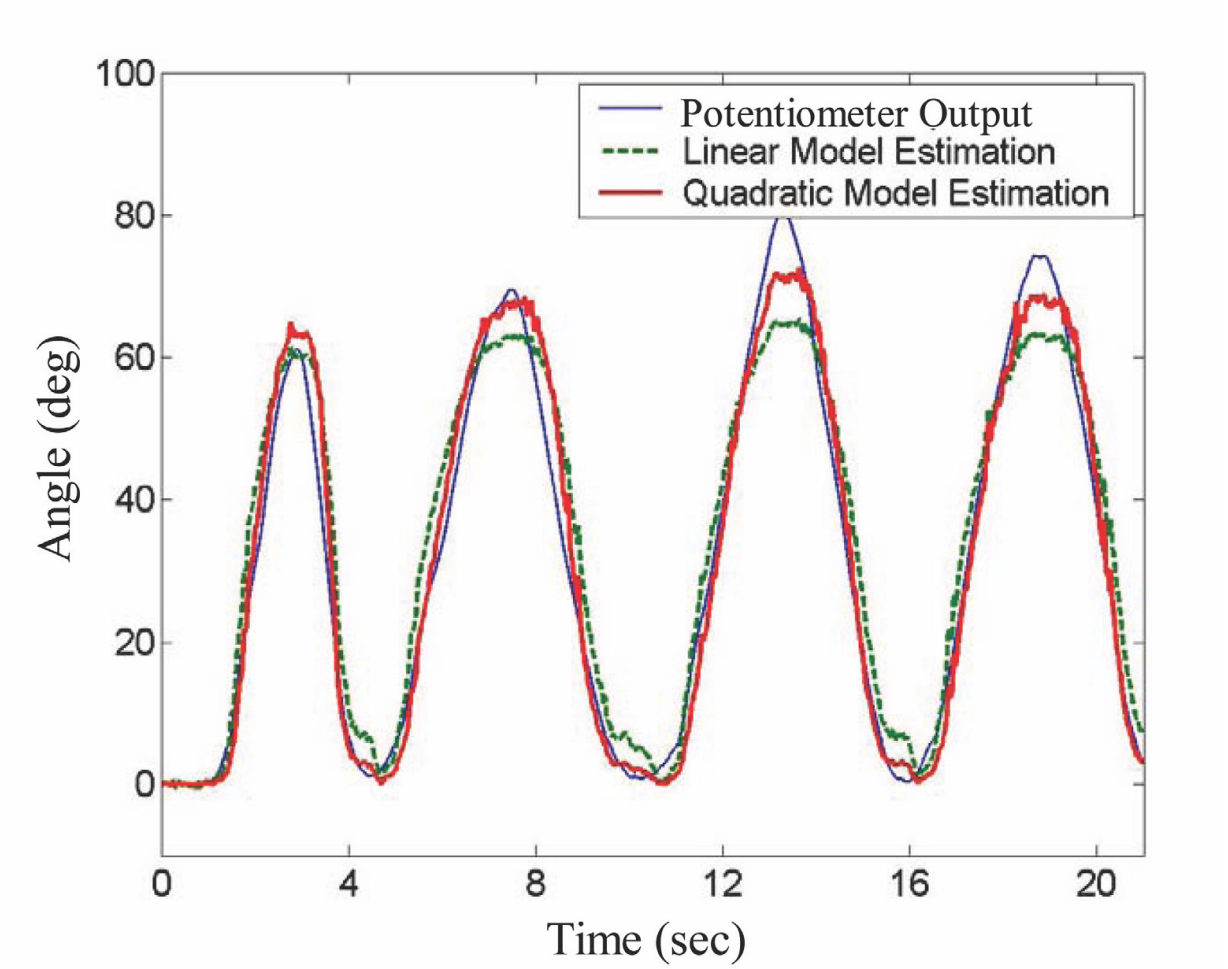
**Sensor outputs **– Comparison of goniometer measured knee joint angle and estimated angles from wearable conductive fiber sensor.

A calibration was performed to find the optimal regression matrix for both the linear and nonlinear predictors, and a sequence of knee movements was then monitored with the sensors. Figure 9 shows the results of a typical sequence of these knee measurements, comparing the estimated angle from the conductive fiber sensors using the predictor models to that of the rotary potentiometer firmly attached to the leg.

The performance of the pants sensors can be seen to be quite good, accurately capturing the joint movement patterns over time. The average rms error between the pants sensor estimate and the potentiometer using the linear predictor was 5.4°, while that for the quadratic predictor was significantly better, at just 3.2°.

**Figure 11:**
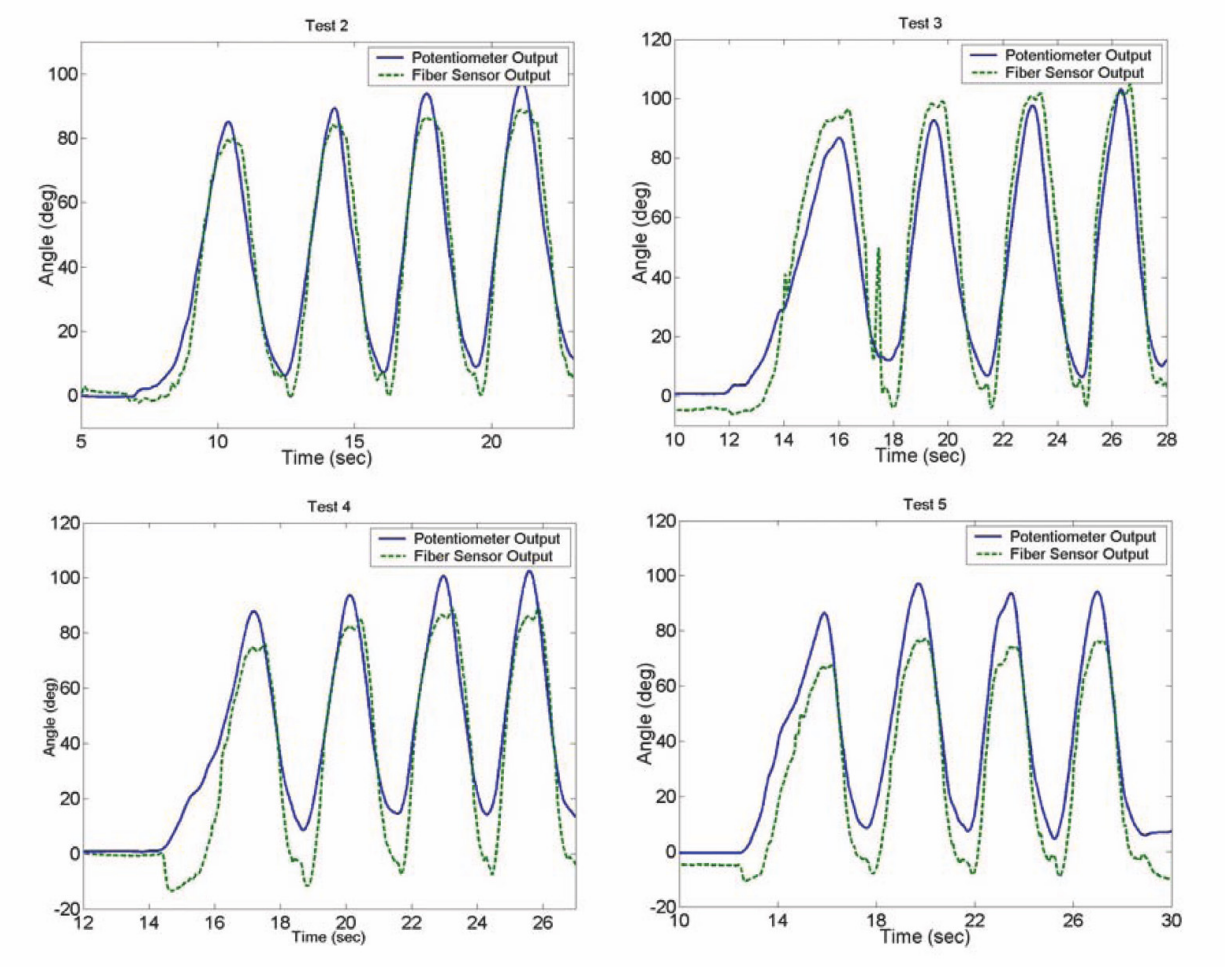
**Self-Registration Results. **Joint angle measurements with sensing garment taken off and put back on before each test.

It is important that these sensors are able to measure all types of motion, including higher frequency motion. To determine the frequency capabilities of the prototype fiber sensors, tests were performed where the leg was swung back and forth at different frequencies. The resulting sensor estimations, and errors when compared to the potentiometer, are summarized in Figure 11 and Table 1 respectively.

**Table 1:** Sensor Frequency Capability Results

*Approximate Frequency (Hz)*	*Average RMS Error (degrees)*
0.1	3.8
0.5	6.6
1	5.5
1.5	4.9
2	7.1

Fiber sensor thread errors for various frequencies of joint motion.

From these results, it is seen that the sensors are able to track the joint motion for frequencies as high as 2 Hz, but significantly larger errors result as the frequency is increased. Since most gross human motion takes place below these frequencies in a typical day, these sensors are suitable for everyday measurements, but such limitations should be considered if more accurate measurements are desired.

Since these sensors are to be worn multiple times by a user, the reliability of registration is important every time the sensors are worn. Therefore, it is important that using the template-matching algorithm with an array of sensors will give an accurate registration each time the sensors are taken off and put back on. To verify this, an initial repeatability test was performed on the prototype sensor pants. The pants sensors were taken off and put back on four separate times to simulate four future uses of the sensors after an initial calibration test. The knee joint was moved over a wide range of motion in each instance. The joint angles measured by the fiber sensors for each test are shown in 11. The errors between these measurements and the potentiometer measurements are summarized in Table 2.

**Table 2:** Sensor Self-Registration Results

*Test Number*	*Average RMS Error (degrees)*
2	5.7
3	8.6
4	8.5
5	11.6

Fiber sensor thread errors for successive tests where pants have been taken off and put back on.

Again, the sensors are able to capture the overall motion of the knee in each case, but appear to give less accurate results each time the pants are worn. For this reason, while a completely self-calibrating sensor is always desirable, it may be necessary to re-calibrate the sensors after many uses for more accurate measurements.

### Double Axis Results

**Figure 12:**
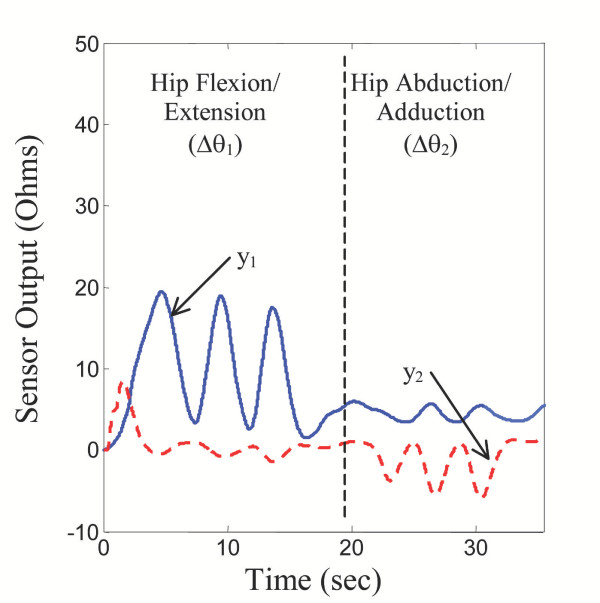
**Multi-Axis Sensor Outputs. **Hip sensor outputs for two distinct leg motions.

Figure 12 shows sensor outputs for a sequence of semi-random leg movements. In this case, output was captured from sensors *y*_1 _and *y*_2_, spanning the posterior and lateral side of the hip, respectively (see Figure 2). In the first segment of motion, the leg was kept fully extended in the sagitall plane, and the subject performed a flexion/extension three times (*θ*_1 _varies, while *θ*_2 _= 0). In the second segment, leg movement was allowed only in the frontal plane, while the subject performed an abduction/adduction movement three times (*θ*_2 _varies, *θ*_1 _= 0).

In each case, the sensor spanning the axis in which the angle changes took place was the most sensitive to change, as expected. Each joint motion also produced small, but not insignificant, cross-coupling outputs in the "remote" sensors as well, showing that a single sensor output is dependent on multiple joint angles, and not one single angle.

The pants sensor threads about the hip joint were then calibrated with the twin-axis goniometer. Table 3 shows the calibration matrix obtained per (9) using the predictor expression of (11). As can be seen, the first-order diagonal terms are dominant, with the cross-coupling terms significant, but not as dominant. The third and higher-order non-linearities were found to be insignificant compared to the values shown, and thus a second order predictor of the form of (11) seemed sufficient.

**Table 3:** Calibration Matrix

	*y*_1_	*y*_2_		
	2.86	0.27	0.04	-0.24
	1.32	3.83	-0.29	0.17

Calibrated parameter matrix for two hip sensor threads on one individual.

**Figure 13:**
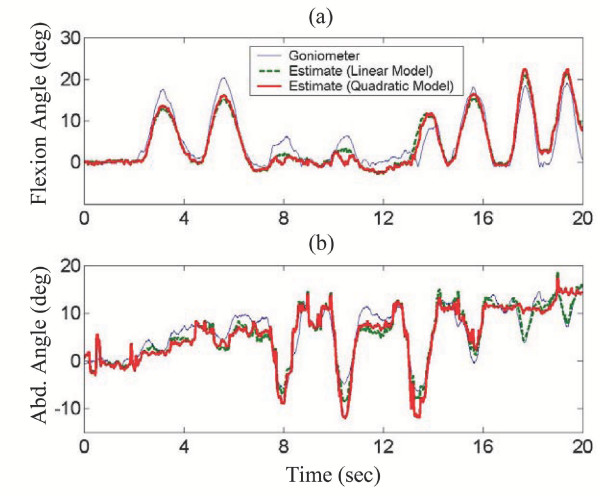
**Hip Joint Measurement Results. **Comparison of goniometer measured hip joint angles and estimated angles from wearable conductive fiber sensors: (a) Hip flexion/extension, (b) Hip abduction/adduction.

After initial calibration, random leg movements were then monitored with the sensors. Figure 13 shows the results of a typical sequence of the resulting hip angle measurements. Again, the estimated angles from the conductive fiber sensors using both a linear and quadratic predictor are compared to that of a twin-axis goniometer.

The pants sensors were again able to capture the joint movement patterns over time, in this case for two axes of motion. The average rms error between the pants sensors' estimate of hip flexion angle and the goniometer's was 2.5° using the linear predictor and 2.4° using the quadratic predictor. For hip abduction, these errors were 2.1° and 1.7° respectively. In this double axis case, the differences between the linear and quadratic predictors were not very significant over the typical ranges of hip joint angles measured.

Previously, the assumption was made for the double axis hip joint that both sensor arrays would be offset from their calibration position by the same amount for each use. This allowed the double axis registration to be reduced to a single axis registration. To verify this assumption, a simple experiment was performed on the pant's hip sensors. The pants were taken off and put back on ten times. Each time, the distance around the waist between the sensor thread on the side of the hip, and the sensor thread on the rear of the hip was measured (distance between Point A and B in Figure 2). The average distance measured on a single individual in this way was 12.5 cm, with a standard deviation of 0.1 cm. The greatest discrepancy between any of these ten measurements was 0.6 cm (Maximum was 12.8 cm, minimum was 12.2 cm), which is approximately the same distance that separated the single threads in the array over the knee joint. Therefore, slight errors may result from making this assumption, but overall these errors should not contribute much due to the small variation in this experimental data.

## Discussion

For continuous joint monitoring, it should be noted that there are at least three fundamental sources of uncertainty in sensor output. The resistance measures across a section of conductive fiber, while ideally linearly related to length, may differ from an expected value due to the following factors: 1) movement of the fiber across the wire contact point may affect sensor output due to uncertainty in the area being contacted, and dynamic effects of the constant rubbing action; 2) although the elastic cord takes up a majority of the sensor tension, slight changes will also take place in the fiber tension as the joint is moved, and this will affect fiber resistance; and 3) different sections of even the same fibers will exhibit slightly different resistance characteristics due to the slightly inhomogeneous nature of such fibers. In spite of all these sources of uncertainty, it is still possible to accurately calibrate a set of sensors, and achieve acceptable joint measurements with minimal errors. These effects are minimized through careful selection of the particular fibers used as sensors, and in manufacturing the garment.

While two specific predictor models have been presented for the calibration of a set of sensors, there are of course many more candidates that could be used as well. The linear and quadratic models used in this paper were the simplest choices, and the experimental results showed no advantage to adding more terms. Doing so only increased the computational requirements unnecessarily. This is why the models were presented as they were.

A few more words should also be said about the registration algorithm. As presented, this algorithm only accounts for shifting of a set of sensors in one direction (particularly, in the "horizontal" direction). It is felt that this is appropriate due to the construction of the sensing garment. With the sensors instrumented in a "vertical" fashion, the user is responsible for visually checking that they put the garment on with no twist. This is relatively easy to do with the fibers oriented vertically. Furthermore, as long as the sensors span well beyond the local effects of skin movement around a joint, small shifts in the vertical direction will theoretically have little to no effect on the sensor output. Requiring a patient to "zero" the sensor output with all joints in the 0° position each time the garment is worn further eliminates any errors due to sensor drift.

Finally, the wearability of the pants sensing garment must be addressed. What makes this sensing garment "more wearable" than existing joint measurement devices is that it is simply a pair of pants that people already wear on a regular basis.

The extra sensors and wires added to these pants are compact and lightweight, almost negligible to the wearer. These sensors are easy to use, requiring much less skill and carefulness by the user, in general, than a typical goniometer.

## Conclusion

A wearable joint movement sensor design has been presented that uses conductive fibers incorporated into a fabric that is form fitting to a joint. Resistance changes in the fibers caused by fiber movement as the joint is moved can be related to angular joint position. Using multiple fiber sensors, multi-axis joint angles can be determined, in addition to single-axis angles, after a one-time calibration procedure performed by a therapist/physician. Implementing a nonlinear predictor model, continuous joint angle measurements can be made during daily activities, with the sensor able to be taken off and put back on at any time with no need for manual recalibration. Sensor offsets due to misregistration can be accounted for through the use of a sensor array spanning the joints of interest. This allows the sensors to self-calibrate, with only a few simple motions of the patient.

After preliminary experiments involving a pants sensing garment for lower body monitoring, it has been seen that this methodology is feasible for monitoring joint motion of the hip and knee. Multiple sensor arrays are used at multi-d.o.f. joints, where each sensor output is coupled to multiple joint angle changes. This design therefore produces a robust, comfortable, truly wearable joint monitoring device. This paper outlines the development of this sensor from initial idea to working prototype. Future effort is needed in developing a completely wearable, highly accurate sensor, though. This would include making the sensors wireless, and therefore "tether-free." More precise textile manufacturing techniques would also be needed to further reduce measurement errors.

## Competing interests

The author(s) declare that they have no competing interests.

## Authors' contributions

PTG developed the ideas discussed in this paper under the guidance of HHA. PTG carried out all experiments. Both authors read and approved the final manuscript.

**Figure 10:**
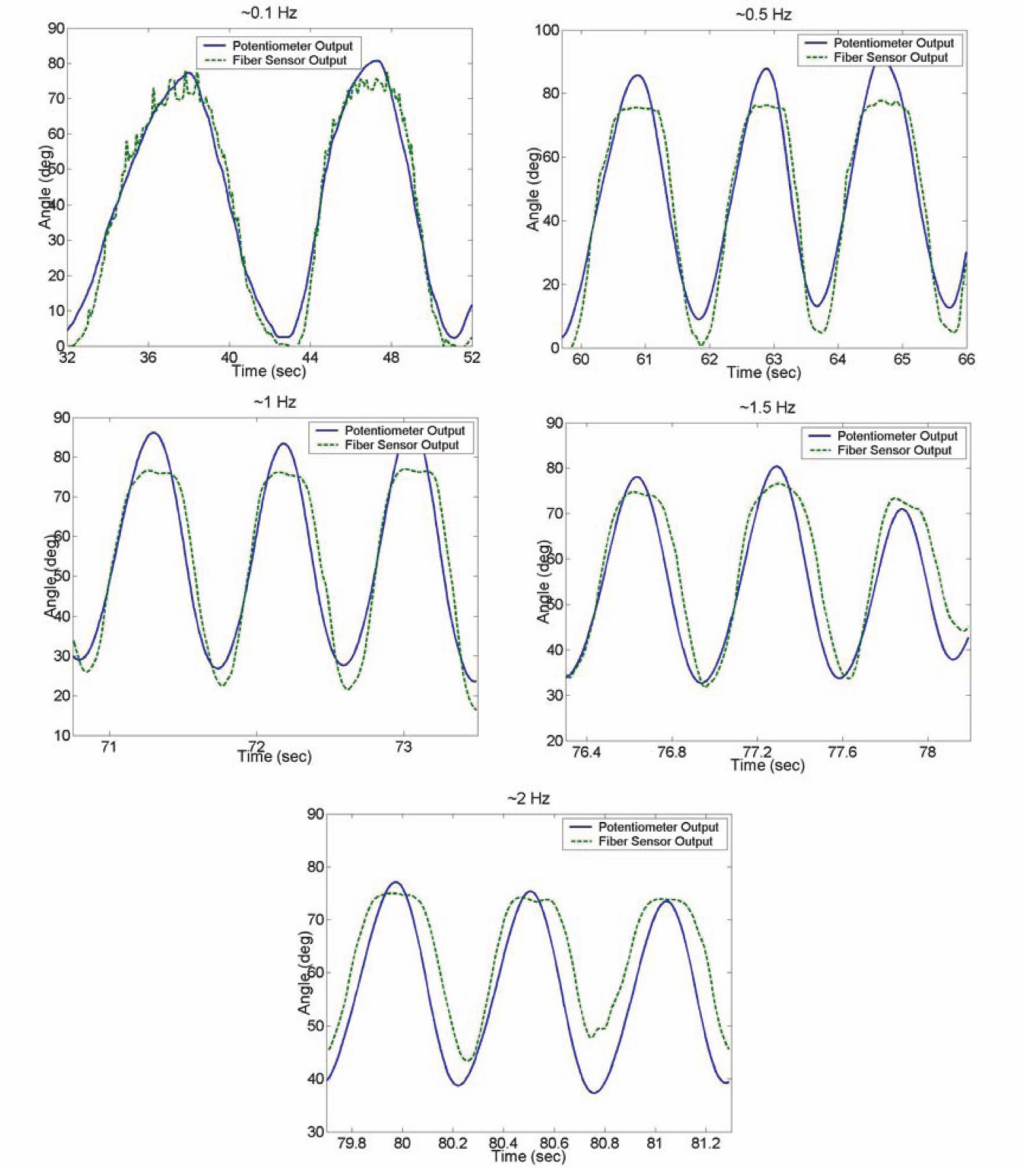
**Frequency Variation Results. **Joint angle estimations for various frequencies of joint motion.
